# YY1 activates EMI2 and promotes the progression of cholangiocarcinoma through the PI3K/Akt signaling axis

**DOI:** 10.1186/s12935-021-02328-6

**Published:** 2021-12-21

**Authors:** Shuai Zhou, Kang Lin Qu, Jin Ang Li, Shi Lei Chen, Yi Gang Zhang, Chao Zhu, Hao Jin, Yong Wang, Qing Pang, Hui Chun Liu

**Affiliations:** 1 Department of Hepatobiliary Surgery, The First Affiliated Hospital of Bengbu Medical College, Bengbu, 233000 Anhui China; 2 The Fourth Department of General Surgery, Second People’s Hospital of Anhui Province, No. 1868 Dangshan Road, North Second Ring, Hefei, 230041 Anhui China

**Keywords:** EMI2, YY1, Cholangiocarcinoma, PI3K/Akt signaling pathway

## Abstract

**Background:**

Cholangiocarcinoma (CCA) is one of the deadliest cancers of the digestive tract. The prognosis of CCA is poor and the 5-year survival rate is low. Bioinformatic analysis showed that early mitotic inhibitor 2 (EMI2) was overexpressed in CCA but the underlying mechanism is not known.

**Methods:**

The data on bile duct carcinoma from TCGA and GEO databases were used to detect the expression of EMI2. The transcription factors of EMI2 were predicted using JASPAR and PROMO databases. Among the predicted transcription factors, YY1 has been rarely reported in cholangiocarcinoma, and was verified using the luciferase reporter gene assay. RT-PCR was performed to predict the downstream pathway of EMI2, and PI3K/Akt was suspected to be associated with it. Subsequently, in vivo and in vitro experiments were conducted to verify the effects of silencing and overexpressing EMI2 and YY1 on the proliferation, invasion, and metastasis of the bile duct cancer cells.

**Results:**

EMI2 was highly expressed in CCA. Silencing EMI2 inhibited the proliferation, invasion, and migration of CCA cells, arrested cell cycle in the G1 phase, and promoted of apoptosis. The luciferase reporter gene assay showed that YY1 bound to the promoter region of EMI2, and after silencing YY1, the expression of EMI2 decreased and the progression of CCA was inhibited. Moreover, key proteins in the PI3K/Akt signaling pathway decreased after silencing EMI2.

**Conclusion:**

EMI2 may be one of the direct targets of YY1 and promotes the progression of CCA through the PI3K/Akt signaling pathway.

**Supplementary Information:**

The online version contains supplementary material available at 10.1186/s12935-021-02328-6.

## Introduction

Due to the increase in incidence, more and more researchers have focused their efforts on understanding cholangiocarcinoma (CCA). Based on the location, CCA is classified into different types [1–3].The pathogenesis of CCA is still unclear, and possible factors include hepatitis B, cholangiolithiasis, hepatic fascidiasis, chronic cholangitis, etc. Lymph node invasion is a key factor in poor prognosis of CCA [2]. Cholestasis and chronic cholangitis are recognized factors in the pathogenesis of CCA [3]. Chronic cholangitis can stimulate the secretion of cytokines and damage the DNA repair function, resulting in the formation of pro-cancer microenvironment, which involves the activation of multiple signaling pathways, such as PI3K/AKT signaling pathway [4]. However, CCA often has no obvious symptoms in the early stage, and only jaundice, fatigue, abdominal pain and other symptoms will appear in the progressive stage. Waiting for treatment, the best treatment opportunity is often missed, and most patients who receive radical surgery have a low 5-year survival and a high recurrence rate [5]. At present, gemcitabine combined with other platinum drugs is the main first-line drug for CCA, but the effect is still poor, and new target drugs need to be developed. Therefore, the study of new prognostic targets is crucial to understand the molecular mechanism of CCA.

Early mitotic inhibitor 2 (EMI2), also known as FBXO43 and XERP1, is a subclass of the F-box protein family, a protein with an F-box domain [6]. EMI2 is an inhibitor of ubiquitin ligase and is involved in inhibiting the formation of late complex/cyclosome (APC/C) [7]. APC/C promotes the activity of cyclin B1, thereby promoting cell cycle progression. EMI2 consists of an N-terminal regulatory region and a C-terminal functional region [8, 9]. The N-terminal domain possesses a binding motif required for the ubiquitin-linked enzyme-dependent degradation of EMI2 [10]. The C-terminal region competitively binds APC/C with cyclin B, and there is also a zinc-binding region at the C-terminal, which inhibits APC/C ubiquitin ligase activity [11]. Studies have reported that EMI2 is overexpressed in hepatocellular carcinoma and associated with a poor prognosis [12]. In addition, the prognosis is also poor in breast cancer patients with a high expression of EMI2 [13]. However, the specific role of EMI2 in CCA is not reported.

Through database detection, we found that YIN-YANG 1 (YY1) is a transcription factor of EMI2. YY1 is a member of the Gli-Kruppel family and is involved in a variety of biological processes, such as cell proliferation, cell cycle progression, and transcriptional regulation [14–17]. YY1 is involved in regulating the progression of bile duct carcinoma. Xu et al. [18] found that Circ-CCAC1 binds miR-514a-5p as a molecular sponge, and miR-514a-5p binds YY1 to promote the progression of bile duct carcinoma. Circ-LAMP1 inhibits miR-556-5p and miR-567 to promote the expression of YY1, thereby promoting the progression of CCA [19]. YY1 activated LINC00667, and LINC00667 played a ceRNA mechanism to participate in the binding of miR-200c-3 to promote the process of epithelial mesenchymal transformation of CCA [20]. YY1 activates PCAT1 to promote the proliferation, invasion, and migration of CCA [21]. At present, the studies on the role of YY1 in CCA are limited to non-coding RNAs, and whether YY1 directly regulates EMI2 to play a regulatory role in CCA, needs to be explored.

In the current study, we analyzed the expression of EMI2 and its upstream and downstream molecules in CCA, to identify potential targets for the diagnosis and treatment of CCA.

## Materials and methods

### Database selection and analysis

Based on the gene expression data in CCA from the The Cancer Genome Atlas (TCGA) database (https://tcga-data.nci.nih.gov/tcga), FPKM data of CCA were downloaded and analyzed for any differences, and the mRNA expression levels of EMI2 were presented using a box diagram. From the GEO database (https://www.ncbi.nlm.nih.gov/geo/) to download GSE32225 (https://www.ncbi.nlm.nih.gov/geo/query/acc.cgi?acc=GSE32225), and GSE22633 (https://www.ncbi.nlm.nih.gov/geo/query/acc.cgi?acc=GSE22633) data processing, and differential genes were screened by limMA package. The TIMER (http://timer.cistrome.org/) was used to check the quantity of EMI2 expressed in various tumors.

PROMO (http://alggen.lsi.upc.es/cgi-bin/promo_v3/promo/promoinit.cgi?dirDB=TF_8.3) and ChIPBase databases (http://rna.sysu.edu.cn/chipbase/) were used to predict the transcription factors of EMI2, then take intersection with Wayne figure, YY1 and STAT4 were found to be mating sites. JASPAR database (http://www.jaspar.genereg.net) was used to predict the possible binding sites of EMI2 and YY1.

### Tissue sample collection

Paired tumor and paracancerous tissue samples were collected from 8 patients with CCA who underwent surgical resection in the First Affiliated Hospital of the Bengbu Medical College from January 2020 to January 2021. The tissue samples were cryopreserved at − 80 °C. All patients signed informed consent, and the study was approved by the Ethics Committee of the First Affiliated Hospital of the Bengbu Medical College.

### Cell culture and transfection

CCA cell lines (RBE, HuCCT1, HUCC-9810, and QBC-939) were purchased from the Shanghai Cell Bank, Chinese Academy of Sciences, and normal bile duct epithelial cell line (HIBEpiC) was purchased from the Shanghai Tongpei Biotechnology Co., Ltd. HuCCT1, RBE, HUCC-9810, QBC-939, and HIBEpiC cells were treated with RPMI-1640 medium (Gibco, USA), 10% fetal bovine serum (FBS, Gibco, USA), and 1% penicillin–streptomycin (Biosharp, China) and incubated at 37.5 °C with 5% CO_2_.

To construct a stable transgenic vector using human EMI2 lentivirus, the single-stranded DNA oligonucleotide was synthesized by RNA interference target sequence technique, and the double-stranded DNA was annealed to form the double-stranded DNA. The restriction sites were 5ʹ-GGATCC(BamHI) and 3ʹ-GAATTC (EcoRI). The double-stranded DNA was inserted into the PLENR-GPH vector (Snap-gene sequencing sequences are shown in Additional file 1: Table S1) of RNAi lentivirus after digestion using multiple restriction enzymes. The interference sequence was 5ʹ-GCGTGAAATTGTTGTTCAAGA-3ʹ and the control sequence was 5ʹ-TTCTCCGAACGTGTCACGT-3ʹ (Shanghai Baioujing Biotechnology Co., Ltd.). The siRNA sequence of YY1 was 5ʹ-CCAAACAACUGGCAGAAUUTT-3ʹ (Shanghai Baioujing Biotechnology Co., Ltd.). The ligation products were then used to transform competent *Escherichia coli* cells, and the recombinants were detected using PCR. Lentiviral vectors were collected 72 h after transfection. Subsequently, the recombinant lentivirus (100 μL, 1 × 10^7^ units/mL) with green fluorescent protein were cultured at a density of 2 × 10^5^ cells/well for 24 h. After 72 h, stable lentiviral strains expressing shCtrl and shEMI2 were obtained. Meanwhile, YY1 and EMI2 overexpression plasmids were constructed using pEGFP-N1 vector. First, YY1 (Gene ID:7528) and EMI2 (Gene ID: 286151) sequences were obtained according to NCBI, and the sequences were synthesized with Shanghai General Biology Company. The target sequence was subcloned into pEGFP-N1 vector according to the designed enzyme cutting site. Subsequently, the recombinant plasmid was amplified into a monoclonal colony, and the plasmid was extracted using a plasmid extraction kit (Beijing Solaibao Biotechnology Co., LTD.).

### RNA extraction and RT-PCR

Total RNA was extracted with Trizol reagent (Invitrogen, USA), and then reverse transcription was performed using the cDNA preparation kit according to the manufacturer’s instructions (Thermo, USA). The primers for all PCR primers, and their internal reference sequences were designed using Primer 5 (Additional file 1: Table S2). Then, quantitative PCR was performed using a real-time PCR machine (MX3000p, Agilent).

### Western blotting

The transfected and control cells were digested using RIPA lysis buffer with a protease inhibitor and the total proteins were extracted. BCA kit (Beyotime Biotechnology, China) was used to detect the protein content. After quantification, the protein samples were subjected to the SDS-PAGE gel electrophoresis separation. Then, the target proteins were transferred to polyvinylidene fluoride (PVDF) membrane, which was sealed with protein-free rapid blocking solution (Beyotime Biotechnology, China) for 30 min. The primary antibody was added and the membranes were incubated at 4 °C overnight. Then use PBST to clean the membrane 3 times. They were imaged after incubation with the Tanon HighsigeCl western blotting substrate reagent for 2 h using a Biorad imaging instrument. The blots were analyzed by Image J software. The antibodies used are shown in Additional file 1: Table S3.

### CCK8 assay

First, RBE and QBC9393 cells were planted in 6-well plates with a cell density of 4 * 104 according to different groups. The cells were treated according to the groups. When the growth reached 80%, the cells were digested and centrifuged, and inoculated into 96-well plates with a cell density of 5000/well. CCK-8 reagent (Beyotime Biotechnology) was added to each well after 1, 2, 3, 4, and 5 days of culture. After adding the CCK-8 reagent, the cells were incubated for 1–2 h at 37 °C. The optical density of cells within each group was detected using a microplate analyzer at 450 nm.

### Tissue microarray (TMA) and immunohistochemistry (IHC)

TMA included tissue samples from 75 patients with bile duct carcinoma (48 patients with extrahepatic bile duct adenocarcinoma, 27 patients with intrahepatic bile duct adenocarcinoma) and five cases had normal intrahepatic bile duct tissue. The microarray chips were purchased from Shanghai Kejie Biological Technology Co., Ltd. IHC antibodies used are shown in Additional file 1: Table S3. The patients’ age, gender, organ, pathology diagnosis, TNM stage, survival time, survival state and clinicopathological grade were noted (Additional file 1: Table S4). After immunohistochemical staining, two pathologists analyzed the results based on the intensity and range of staining using a previously described method [22]. The scores given by the pathologists were 0, no staining; 1, light yellow; 2, brown-yellow; and 3 brown for the standards. Positive cell percentage standards were 0–10%, 0 points; 10–25%, 1 point; 26–50%, 2 points; 51–75%, 3 points; and 76–100%, 4 points. The final result was calculated as the number of positive cells multiplied by the staining intensity.

### Cell cloning

The cells from each treatment group were inoculated in 6-well plates with an inoculation density of 1000 cells/well and a medium containing 10% FBS was added. The cultures were terminated after 2 W. After removing the supernatant, the cells were washed with PBS thrice and 4% paraformaldehyde was added for 15 min for fixing the cells. The cells were then washed with PBS twice. After staining with crystal violet for 15 min, the cells were washed with PBS twice.

### Transwell and wound-healing assays

#### Migration assay

Cells from each treatment group were inoculated in a transwell chamber containing a serum-free medium separately at a density of 1 × 10^4^ cells/well. After 24 h, the supernatant was removed, the cells were washed with PBS thrice, fixed with 4% paraformaldehyde, stained with crystal violet, and imaged. Six visual fields/holes were randomly selected and cells were counted for subsequent analysis.

#### Invasion assay

Firstly, 40 uL matrix glue (diluted in 1:8 medium) was added to the upper chamber of Transwell chamber and fixed in an incubator at 37 °C for 1 h. The cells from each treatment group were inoculated in a serum-free medium separately with a density of 1 × 10^4^ cells/well. Then culture for 48 h, fixed with 4% paraformaldehyde, and stained with crystal violet. Six visual fields were selected to photograph and count the cells of each group.

#### Wound-healing assay

Cells from each treatment group were inoculated in 6-well plates. When the cell density was about 80%, scars were made with 200-uL spears, added 2 mL/well medium containing 2% fetal bovine serum, and photographed at 0, 24, and 48 h.

### Luciferase reporter assay

The binding sites of EMI2 and YY1 were predicted using JAPAR data, and the first three significant hits were selected to construct the plasmids. All vectors were verified by sequencing them. The constructed plasmid was transfected into 293 T cells in 96-well plates. After transfection for 48 h, the medium was removed and the cells were washed once with PBS. After adding the lysate, pre-mixed LAR II was added and the 96-well plates were placed on a shaker at room temperature for 15 min. Then luciferase activity was detected.

### Cell cycle and apoptosis assays

The cells from each treatment group were inoculated in 6-well plates with a density of 30 × 10^4^ cells/well. After 24 h of culture, the cells were digested using trypsin (without EDTA). The cells were centrifuged at 1000 rpm for 10 min, collected, and washed with PBS twice. The centrifugation was continued for 10 min (at 1000 rpm), and the reagents from the APC single-dye apoptosis kit (Beyotime Biotechnology) were added successively according to the manufacturer’s instructions. The samples were tested by flow cytometry. After digestion and centrifugation, the cells from the different treatment groups were fixed with 70% ethanol at 4 °C for 12 h. Then the cells were washed with PBS once. After centrifugation, reagents from the cell cycle kit (Beyotime Biotechnology) were successively added according to the manufacturer’s instructions.

### Animals

Four-week-old BALB/c female nude mice were purchased from Shanghai Lingchang Biotechnology Co., Ltd. The ethics approval to perform the animal experiments was obtained from the Bengbu Medical College. The QBC939 cells of stable transfected strains shEMI2/shCTRL were injected 1 cm into the armpit of the mice. Three weeks after the injection, the mice were sacrificed by neck removal under anesthesia, and the tumors were removed. The tumors were embedded in paraffin and immunohistochemical analysis was performed.

### Data statistics

Statistical analyses were performed using Prism 8.0 (GraphPad Software). Data are expressed as mean ± standard deviation ($\overline{x}$ ± s), and the differences between groups were analyzed using independent t-tests or one-way analyses of variance. *P* < 0.05 was considered statistically significant. Bioinformatic analysis was performed using R software (version 4.0). All experiments were repeated three times.

## Results

### EMI2 was overexpressed in CCA

**Fig. 1:**
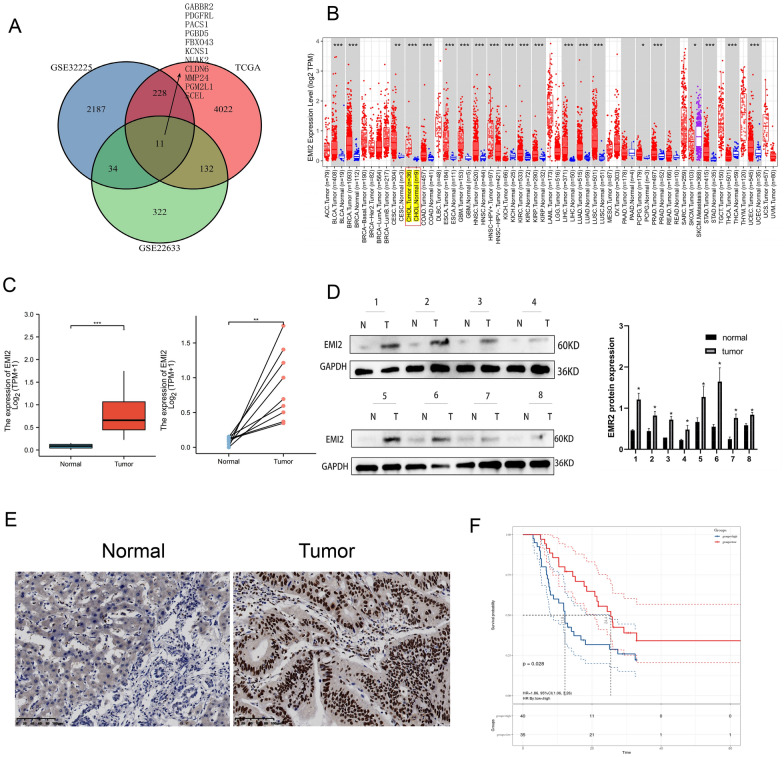
Early mitotic inhibitor 2 is overexpressed in cholangiocarcinoma. **A** The Venn diagram presents the overlap of the significantly upregulated genes (SUGs) between the GSE32225, GSE22633, and The Cancer Genome Atlas (TCGA) datasets; **B** The expression levels of EMI2 in various cancers in the TIMER database; **C** Paired and unpaired analysis of EMI2 based on TCGA database; **D** Western blotting was used to detect the expression of EMI2 in eight pairs of cholangiocarcinoma tissues; **E** Immunohistochemical analysis of tissue microarray revealed that EMI2 was upregulated in bile duct carcinoma; **F** Survival curves were grouped according to the expression levels of EMI2, in which red indicates the low-expression group and blue indicates the high-expression group (*P < 0.05; **P < 0.01; ***P < 0.001)

We combined the TCGA database with GSE3325 and GSE22633 datasets from GEO to search for differentially expressed genes, among which TCGA showed 4393 upregulated genes, GSE3325 showed 2460 upregulated genes, and GSE22633 showed 499 up-regulated genes (Additional file 2: Fig. S1A, B). Out of these, 11 upregulated genes were common to all three datasets (Fig. 1A). We found that the association between EMI2 (FBXO43) and cancer was not common in the literature. In the TIMER database, paired and unpaired analyses of the RNA sequencing files of CCA in the TCGA library showed that EMI2 was overexpressed in CCA (Fig. 1B, C). Subsequently, 8 pairs of CCA tissue samples (5 pairs of intrahepatic CCA and 3 pairs of extrahepatic CCA) were subjected to western blotting to detect the protein levels of EMI2. Compared to paracancerous tissues, EMI2 was highly expressed in the cancer tissues (Fig. 1D). IHC was used to detect the expression of EMI2 in TMA (Additional file 2: Fig. S1C), and the results showed that EMI2 was overexpressed in the cancer tissues (Fig. 1E). Results from TMA also showed that high expression of EMI2 correlated with higher N grade (*P* = 0.047) and M grade (*P* = 0.038) in CCA patients (Table 1). The above results showed that EMI2 was overexpressed in CCA.

**Table 1:** Relationship between tissue baseline data and EMI2 expression level in 75 patients with cholangiocarcinoma

Characteristics	Case	EMI2 expression		*P* value
		Low	High	
All cases	75	35	40	
Age (years)				*P *= 0.382
< 60	43	17	24	
≥ 60	32	18	20	
Gender				*P *= 0.555
Male	39	18	21	
Female	36	17	19	
T grade				*P *= 0.35
T1 + T2	40	20	20	
T3 + T4	35	15	20	
N grade				***P *= 0.047**
N0	58	22	36	
N1–N3	17	6	11	
M grade				***P *= 0.038**
M0	70	35	35	
M1	5	0	5	
Pathology grade				P = 0.171
1-2	48	24	24	
3-4	23	8	15	
Unknown	4	3	1	

### EMI2 promoted the proliferation of CCA cells

**Fig. 2:**
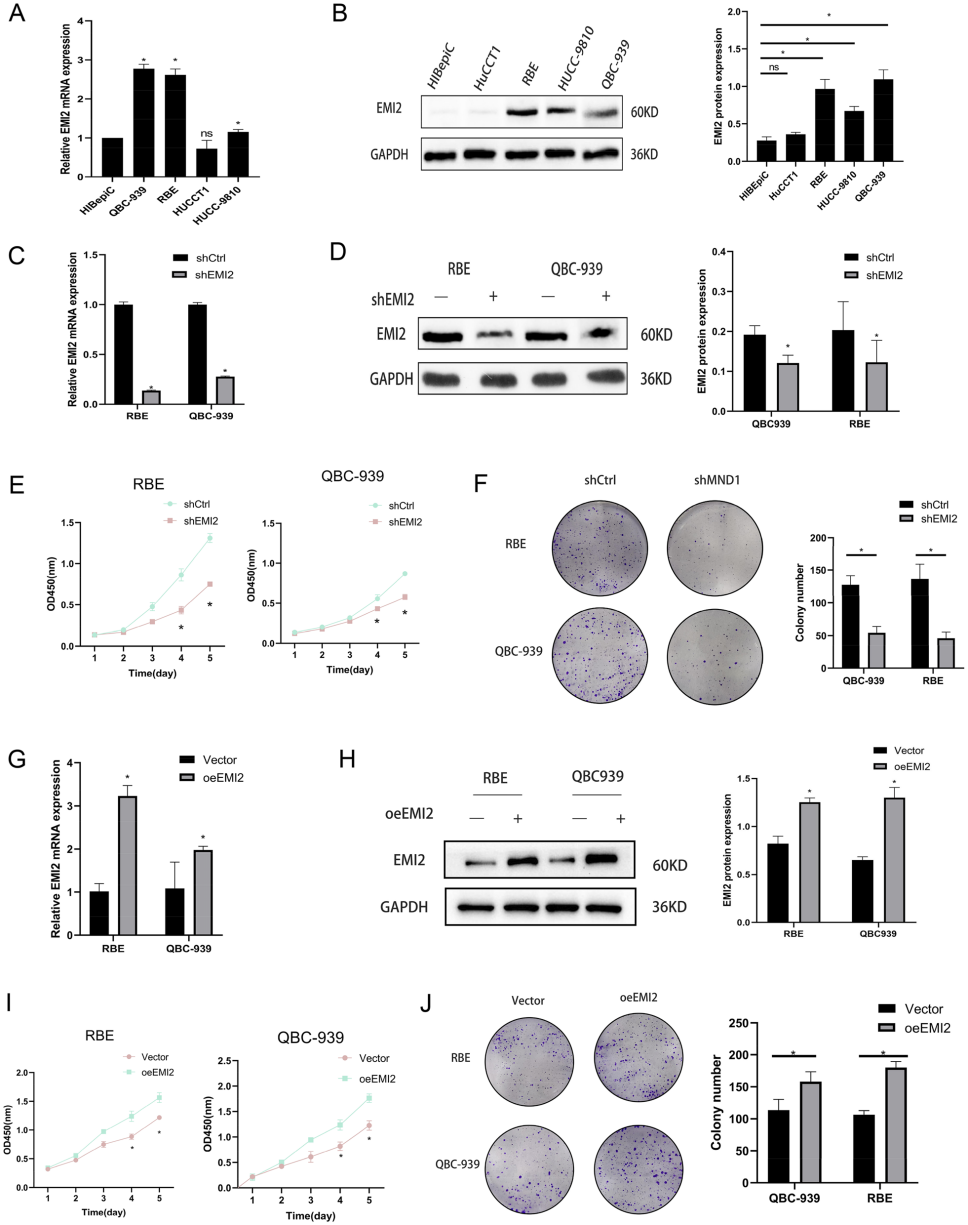
EMI2 promotes the proliferation of cholangiocarcinoma cells. **A**, **B** The levels of EMI2 in healthy intrahepatic bile duct epithelial HIBepic and cholangiocarcinoma cells (QBC939, RBE, HUCCT1, and HUCC-9810) were detected using qRT-PCR and western blotting; **C** qRT-PCR was used to detect EMI2 silencing efficiency; **D** Western blotting was used to detect EMI2 silencing efficiency; **E** CCK-8 assay was used to detect OD450 in each group; **F** Cell cloning test was used to detect the proliferation effect; **G**, **H** qRT-PCR and western blotting were used to detect the overexpression of EMI2. **I**, **J** CCK-8 and colony cloning assays were performed to detect the proliferation of bile duct cancer cells with overexpressed EMI2 (*P < 0.05)

Compared to the healthy intrahepatic bile duct epithelial HIBePic cells, EMI2 was overexpressed in bile duct cancer cell lines, and the highest expression was observed in the QBC939 and RBE cells (*P* < 0.05, Fig. 2A, B). Fluorescent images of lentiviral vector infection are shown in Additional file 2: Fig. S2. The silencing efficiency of EMI2 was detected using qRT-PCR and western blotting, and the difference was statistically significant (*P* < 0.05, Fig. 2C, D). Results of the CCK-8 assay showed that silencing of EMI2 inhibited the proliferation of QBC939 and RBE cells, especially on the 4th and 5th days (*P* < 0.05, Fig. 2E). Colony cloning experiment showed that the number of cell colonies in shEMI2 group was smaller (*P* < 0.05, Fig. 2F). The overexpression efficiency was verified in the recovery experiment (Fig. 2G, H). CCK-8 and colony cloning experiments showed that overexpression of EMI2 promoted the progression of QBC939 and RBE cells (Fig. 2I, J). These results suggested that EMI2 promoted the proliferation of CCA cells.

### The effect of silencing EMI2 on the cell cycle and apoptosis in CCA

**Fig. 3:**
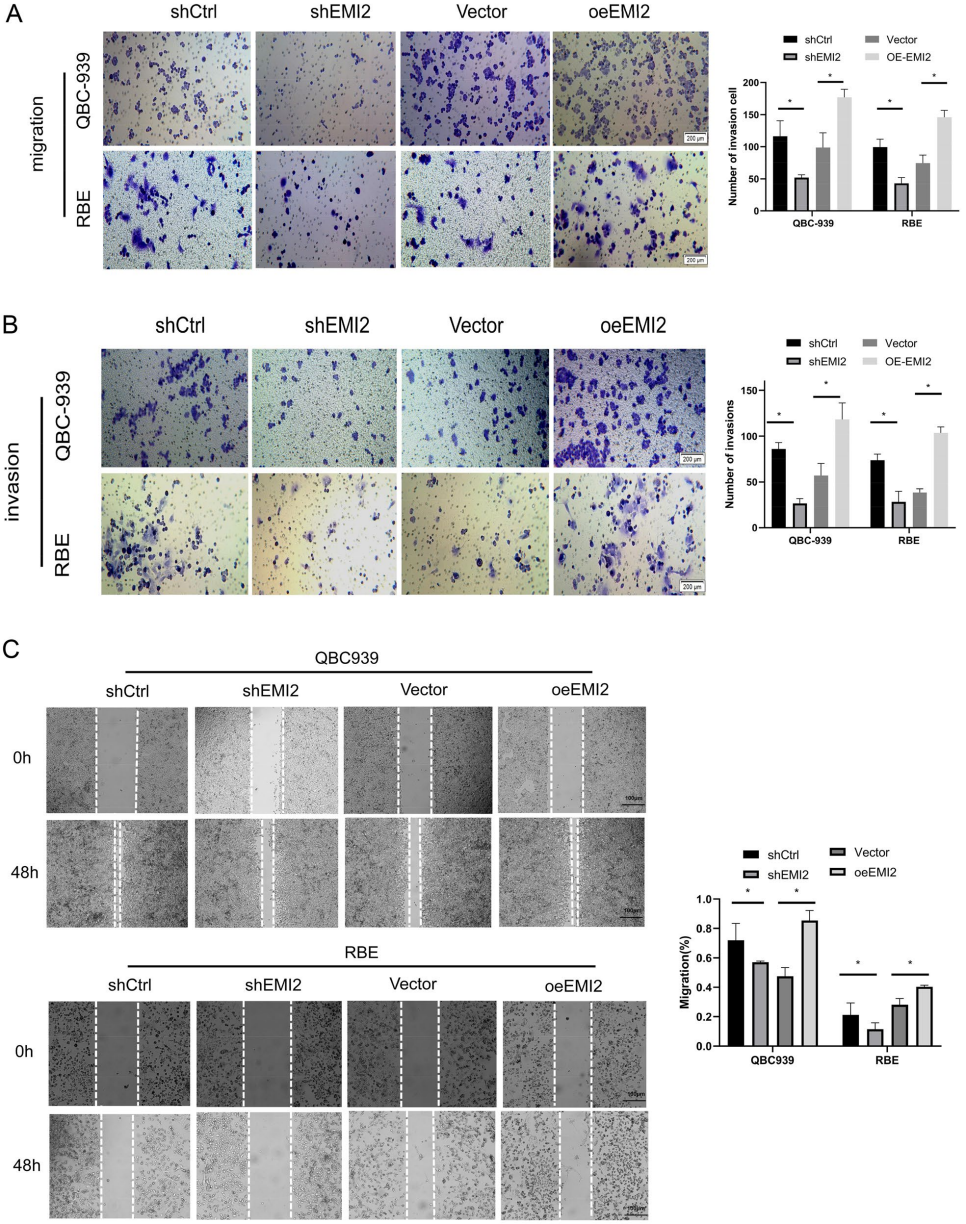
The effects of silencing and overexpressing EMI2 on the invasion ability of the bile duct cancer cells. **A**, **B** Transwell assay verified the changes in the migration abilities of shCtrl, shEMI2, Vector, and oeEMI2 groups; **C** Wound-healing experiment was performed to verify the cell migration rate of the cells in the four groups (*P < 0.05)

**Fig. 4:**
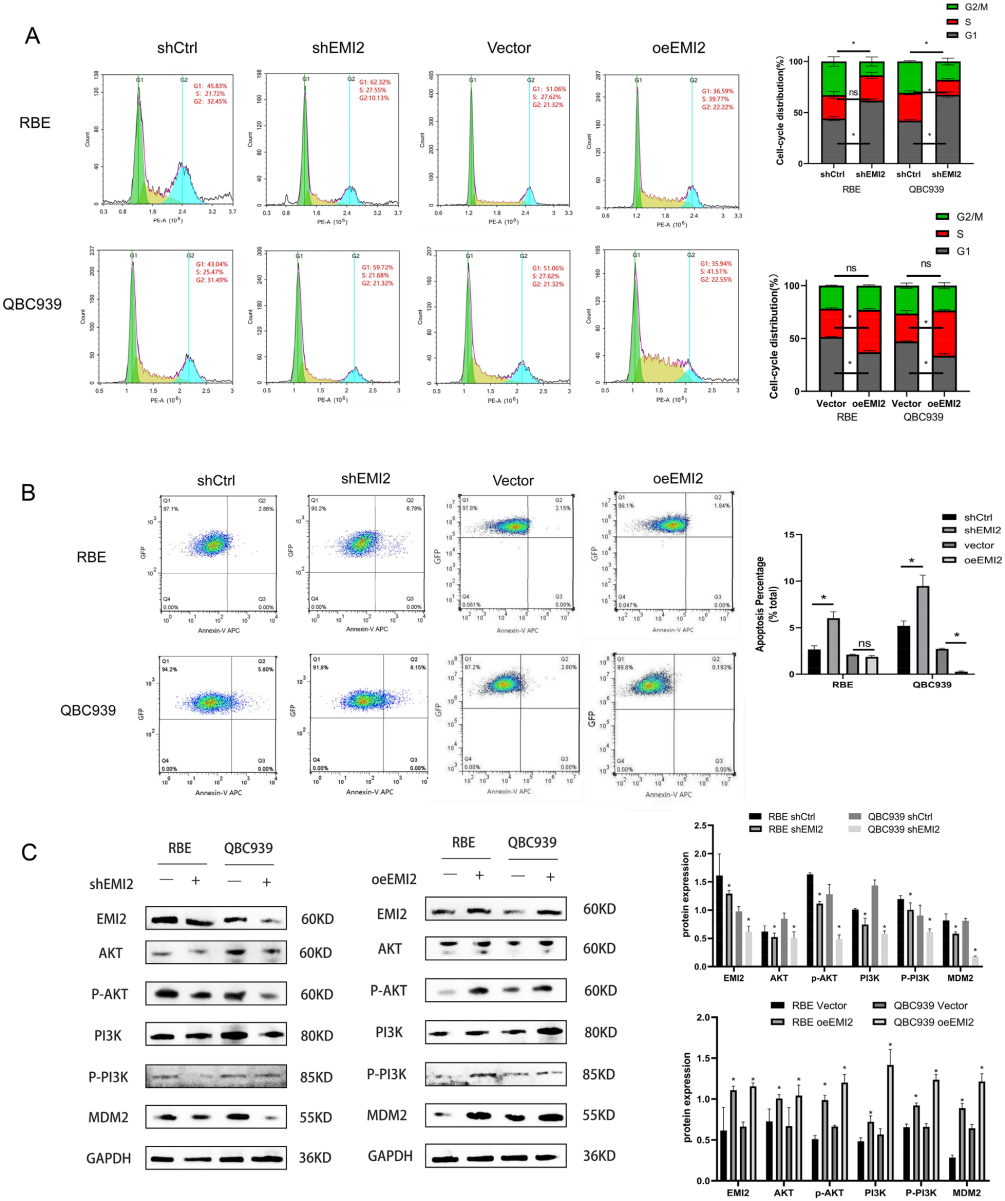
EMI2 promotes cell cycle progression and inhibits apoptosis in bile duct cancer cells. **A** Cell cycle progression in shCtrl, shEMI2, Vector, and oeEMI2 groups was detected using flow cytometry; **B** Annexin-V APC single staining was used to detect early apoptosis in cells in the four groups; **C** Protein expression levels of EMI2, PI3K, p-PI3K, Akt, p-Akt, and MDM2 were detected using western blotting (*P < 0.05)

To determine whether EMI2 promotes the invasive ability of bile duct cancer cells, we performed the transwell assay. The results showed that compared to the shCTRL group, the invasion and migration abilities of the shEMI2 group decreased. After the overexpression of EMI2, the invasion and migration abilities of the oeEMI2 group increased (Fig. 3A, B). The wounding-healing experiment verified the changes in mobility of shCtrl, shEMI2, vector, and oeEMI2 groups, and the mobility decreased after silencing EMI2 and increased after overexpressing it (Fig. 3C). The cell cycle changes in the four groups were detected using flow cytometry, and the results showed that after silencing EMI2, the number of cells arrested at the G1 phase increased. Using the recovery experiment, it was found that overexpression of EMI2 decreased the number of cells arrested at the G1 phase (Fig. 4A). Subsequently, Annexin-V APC single staining technique was used to detect the apoptotic cells in the four groups, and the results showed that the early apoptosis rate increased after silencing EMI2, while the early apoptosis rate decreased after overexpressing EMI2 (Fig. 4B). To further predict the signaling pathway employed by EMI2, EMI2 was silenced in the RBE cell, and qRT-PCR was performed to detect the effects of silencing EMI2 on the mRNA levels of 18 star molecules, among which CDKN1B, P53, and TNF-a were overexpressed, while mTOR and MYC were downregulated. CDKN1B and mTOR were the most significantly downregulated and this result was further confirmed using western blotting (Additional file 2: Fig. S3A, B). CDKN1B and mTOR are key downstream molecules in the PI3K/Akt signaling pathway; therefore, we speculated that EMI2 may affect the PI3K/Akt signaling pathway. To further verify the relation between EMI2 and the PI3K/Akt signaling pathway, we performed western blotting to detect the expression of PI3K, p-PI3K, Akt, p-Akt, and MDM2, the key proteins of the PI3K/Akt signaling pathway, after silencing and overexpressing EMI2 separately. The results showed that the expression levels of PI3K, p-PI3K, AKT, p-AKT, and MDM2 decreased after silencing EMI2, and the result was verified using the recovery experiment (Fig. 4C, D). In summary, the results suggested that EMI2 inhibited apoptosis in the bile duct cancer cells and promoted cell cycle progression through the PI3K/Akt/mTOR signaling pathway.

### EMI2 promoted the proliferation of CCA in vivo

**Fig. 5:**
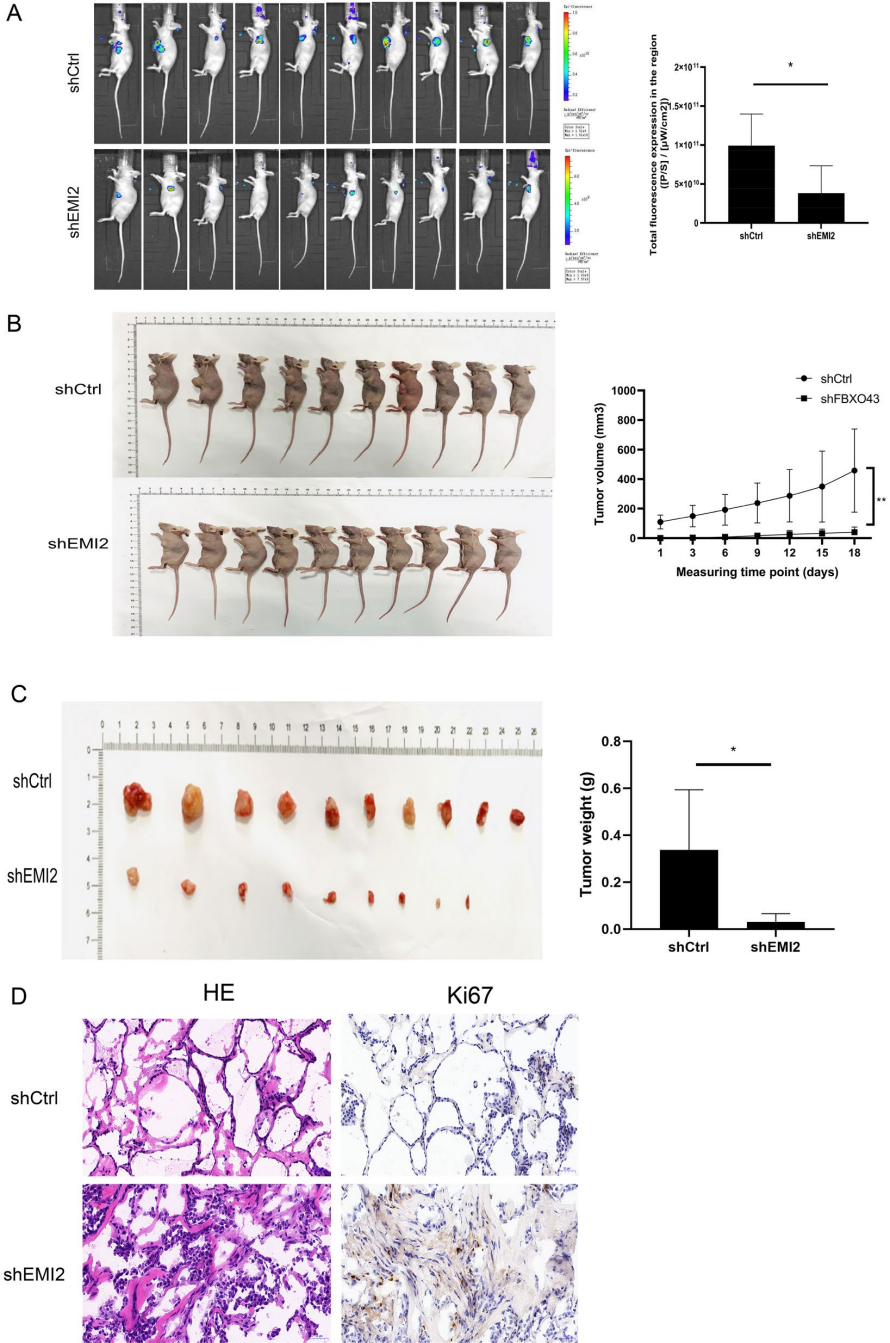
Silencing EMI2 inhibits the progression of bile duct cancer cells in vivo. **A** The detection of subcutaneous tumor formation and fluorescence in nude mice using small animal live imager; **B** Subcutaneous tumor growth curve in nude mice; **C** Subcutaneous tumor images and tumor weight of nude mice; **D** The expression of HE and Ki67 in the tissues of nude mice from the two groups were detected immunohistochemically (*P < 0.05; **P < 0.01)

To further confirm the function of EMI2, tumorigenesis experiments were performed in nude mice. First, transfected shCtrl and shEMI2 cells were subcutaneously injected to establish a nude mouse model. The fluorescence intensity of subcutaneous tumor in nude mice was detected by small animal living imager. Fluorescence measurement results showed that there was a significant difference between the two groups (*P* < 0.05, Fig. 5A). The subcutaneous tumors in the nude mice before dissection were imaged and the tumor growth curve was measured, which showed a statistically significant difference between the two groups (*P* < 0.05, Fig. 5B). After sacrificing the mice, the tumor weight was measured and the results showed that the mice in the shCtrl group (0.338 ± 0.256 g) had significantly larger tumors (*P* < 0.05) than those in the shEMI2 group (0.031 ± 0.035 g) (Fig. 5C). The expression levels of Ki67 in the tumor tissues of the two groups of mice were detected immunohistochemically, and the results showed that silencing EMI2 reduced the levels of Ki67 (Fig. 5D). These results suggested that silencing EMI2 reduced the growth of the bile duct cancer cells in mice, which further confirms that EMI2 promotes the progression of CCA cells.

### YY1 transcription factor activated EMI2 and promoted the proliferation of CCA cells

**Fig. 6:**
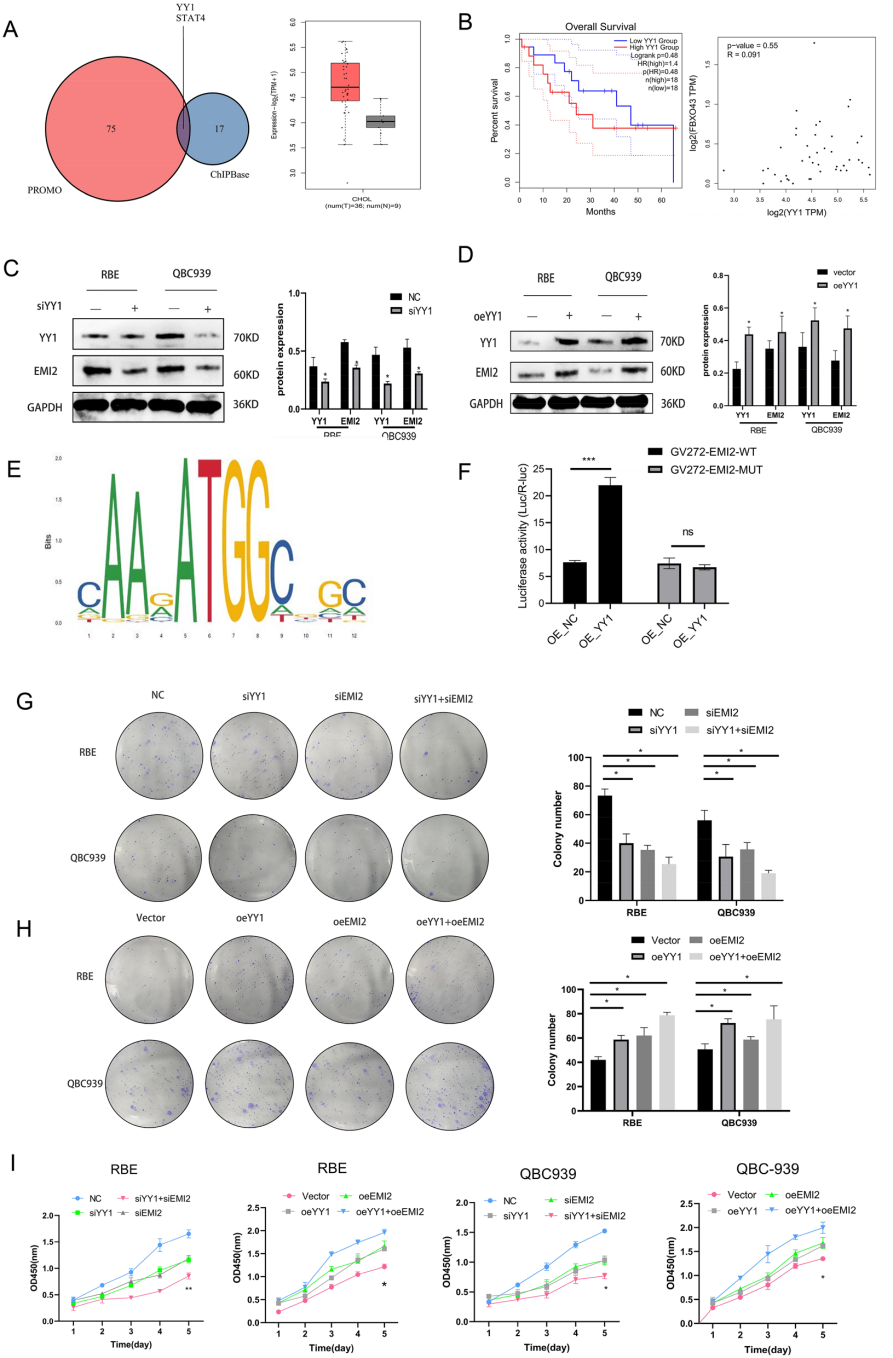
YY1 activates EMI2 and promotes the proliferation of CCA cells. **A** PROMO and ChIPBase databases were used to identify YY1 and STAT4 as the transcription factors of EMI2, and YY1 was found to be differentially expressed in cholangiocarcinoma; **B** The OS curve of YY1 and the correlation between YY1 and EMI2 based on TCGA database; **C**, **D** The protein levels of YY1 and EMI2 after siYY1 and oeYY1 detected using western blotting; **E** A diagram showing the binding sites of YY1 and EMI2 using JASAR database; **F** The effect of YY1 on the fluorescence of GV272-EMI2-WT and GV272-EMI2-MUT was tested using dual-luciferase reporter gene assay; **G**, **H** Cell cloning was used to detect the proliferation in siYY1, siEMI2, siYY1 + siEMI2, oeYY1, oeEEMI2, and oeYY1 + oeEMI2 groups. **I** CCK-8 assay was used to detect the proliferation of cells in each group (*P < 0.05; **P < 0.01; ***P < 0.001)

To further study the regulation of EMI2, the upstream transcription factors of EMI2 were detected using PROMO (n = 75) and ChIPBase (n = 17) databases, and the common transcription factors found were YY1 and STAT4. TCGA data showed that YY1 was highly expressed in bile duct carcinoma (Fig. 6A). However, the overall survival of YY1 based on the TCGA database suggested that high and low YY1 was not correlated with the prognosis of CCA, and the combination of YY1 and FBXO43 was shown based on the TCGA database (Fig. 6B). Western blotting results showed that the expression of EMI2 decreased after silencing YY1, while the expression of EMI2 was restored after YY1 was overexpressed (Fig. 6C, D). The JASPAR database was used to predict the binding sites of YY1 and EMI2, the top three significant hits were selected (Additional file 2: Fig. S3C), and the sequence diagram of the binding sites was downloaded (Fig. 6E). Based on these results, we suspected that YY1 binds to the transcription initiation point of EMI2 and regulates the expression of EMI2. To verify whether YY1 binds EMI2, a dual-luciferase reporter gene assay was performed. The plasmids GV272-EMI2-WT and GV272-FBXO43-MUT were constructed (the mutation sites and base sequences are shown in Additional file 1: Table S5). The dual-luciferase reporter gene assay results indicated that compared to the negative control group, the wild-type oeEMI2 plasmid was significantly positively correlated with the expression of oeYY1, while the mutant plasmid showed no significant difference (Fig. 6F). The experiment was performed using eight different groups—NC (blank plasmid), siYY1 (siYY1 interference), siEMI2 (EMI2 interference), siYY1 + siEMI2 (co-transfected with YY1 and EMI2 interference), vector (invalid transfection sequence), oeYY1 (YY1-overexpressing plasmid), oeEMI2 (EMI2-overexpressing plasmid), and oeYY1 + oeEMI2 groups (co-transfected with YY1 + EMI2 plasmid). Cell cloning and CCK-8 assay were performed to detect the effects on the proliferation of CCA cells. The results showed that compared to the NC group, the bile duct cancer cells in the siYY1, siEMI2, and siYY1 + siEMI2 groups decreased. Out of these, the effect of siYY1 + siEMI2 was the most significant. The results were confirmed using an overexpression experiment (Fig. 6G–I). Meanwhile, we also further verified HUCCT1 and HIBEpiC cells with low EMI2 expression. Western blot, CCK8 and cloning experiments further confirmed the above hypothesis (Additional file 2: Fig. S4). These results suggested that YY1 activates EMI2, thereby, promoting the progression of CCA cells.

### YY1 activated EMI2 to regulate metastasis, cell cycle, and apoptosis in CCA

**Fig. 7:**
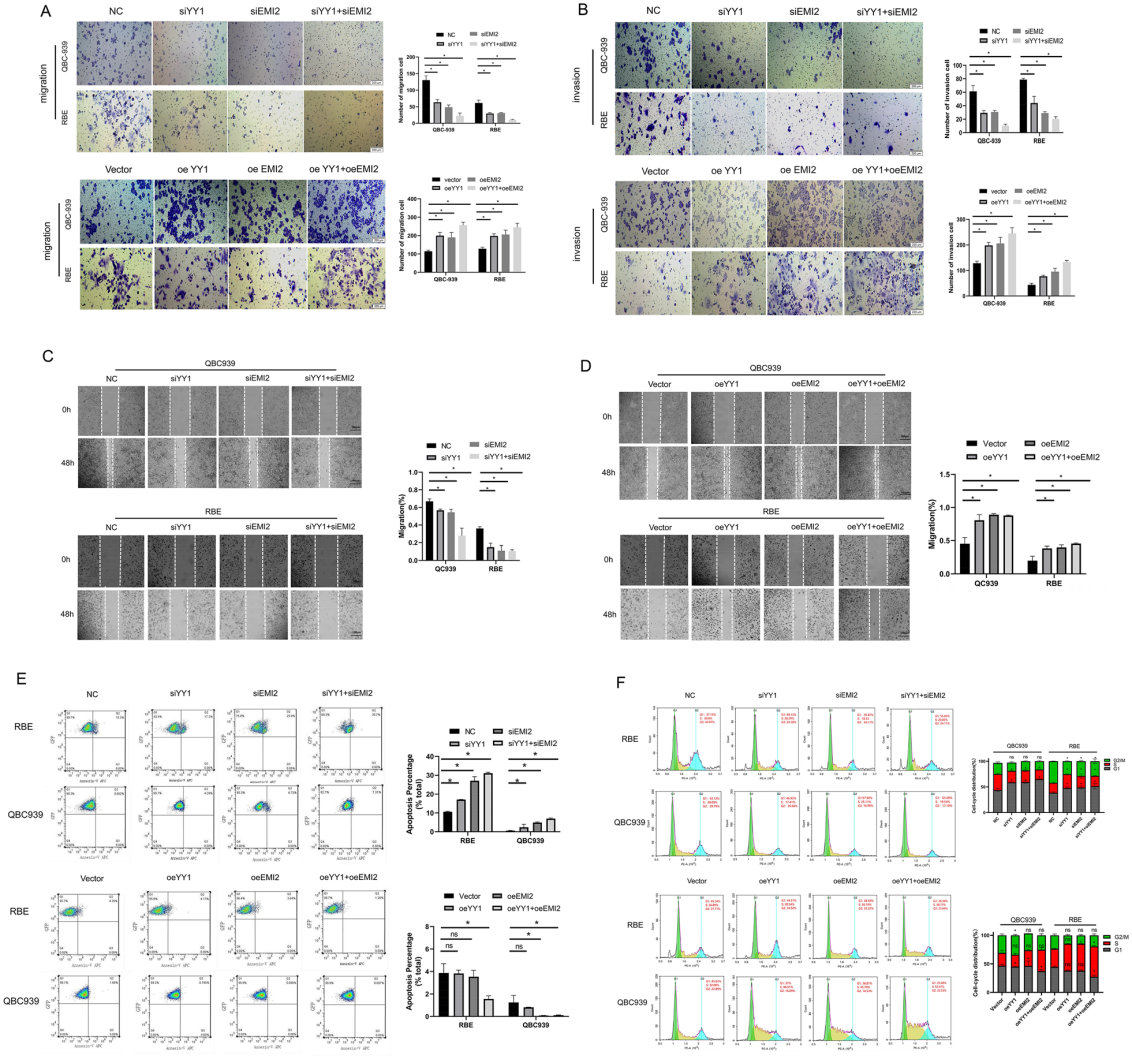
YY1 promotes metastasis and cell cycle progression and inhibits apoptosis in bile duct cancer cells through EMI2. **A**, **B** Transwell assay was used to verify the changes in invasion and migration in each group; **C**, **D** A wound-healing assay was performed to detect the changes in cell mobility in each group; **E** The changes in apoptosis in each group were detected using Annexin-v APC; **F** Cell cycle changes in each group were detected using flow cytometry (*P < 0.05; **P < 0.01; ***P < 0.001)

To further explore the combined regulation of metastasis, cell cycle progression, and apoptosis in bile duct carcinoma by YY1 and EMI2, transwell assay was performed to detect the migration and invasion of cells in each group. The results suggested that silencing YY1 and EMI2 together reduced the ability of the CCA cells to metastasize, and the effect of the siYY1 + siEMI2 group was the highest (*P* < 0.05). This result was confirmed using the overexpression experiment (Fig. 7A, B). We further verified it using the scratch assay, and the results showed that silencing YY1 and EMI2 inhibited the migration of the CCA, and the co-transfection YY1 + EMI2 showed the most significant effect. The overexpression of YY1 and EMI2 together promoted the migration of CCA cells (Fig. 7C, D). Flow cytometry was used to detect the effects of silencing and overexpressing YY1 and EMI2 on apoptosis in the bile duct cancer cells, and the results showed that YY1 activated EMI2, thus, inhibiting apoptosis (Fig. 7E). The cell cycle changes in each group were detected using flow cytometry, and the results indicated that compared to the NC group, the number of CCA cells in the G1 phase increased after YY1 and EMI2 were silenced, while the number of CCA cells in the S phase increased after YY1 and EMI2 were overexpressed (Fig. 7F). These results suggested that YY1 activates EMI2, promotes metastasis, and inhibits apoptosis in CCA cells. The silencing of YY1 also inhibited the expression of EMI2, arresting the CCA cells in the G1 phase.

## Discussion

Dysregulation of the cell cycle is one of the key factors for poor prognosis in cancer, and cell cycle regulation is a complex process [23]. Cell cycle checkpoints play a key role in the regulation of proliferation in CCA. Cell cycle inhibitors, such as CDK-4/7 and other molecules, have also been investigated [24–26]. As an inhibitor of APC/C, EMI2 is involved in the regulation of cell cycle progression. Therefore, studying the underlying mechanism employed by EMI2 is important to further understand the pathophysiology of bile duct carcinoma. In the current study, we first studied the biological functions of EMI2 in CCA. The results of the bioinformatic analysis suggested that EMI2 was highly expressed in CCA; therefore, we speculated that EMI2 might be a potential prognostic molecule of CAA. Studies have reported that EMI2, as a molecule associated with poor prognosis of breast and liver cancer, is involved in the regulation of cancer progression [12, 13]. In our study, EMI2 was overexpressed in patients with CCA, and also in the bile duct cancer cell lines. TME microarray data showed that EMI2 was highly expressed in CCA tissues, and the high expression of EMI2 correlated with the N- and M-staging. This suggested that EMI2 may be one of the molecules responsible for the poor prognosis in CCA.

Cell cycle checkpoints play a key role in the regulation of cell proliferation, and cyclin B is a key factor in the regulation of the transition from the G2 phase to the M phase [27, 28]. Studies have reported that EMI2 inhibits APC/C to stabilize the expression of cyclin B, thus affecting the progression of the cell cycle [7, 29]. In the current study, it was found that after silencing EMI2, the cell cycle was arrested in the G1 phase and early apoptosis was observed. The results from our study also showed that silencing EMI2 inhibited the metastatic ability of bile duct cancer cells. We found that EMI2 promoted the proliferation of CCA cells in vivo. However, whether EMI2 promoted the metastatic ability of CCA cells in vivo is yet to be confirmed. To verify the regulation of EMI2, we used bioinformatic methods to find the upstream transcription factors of EMI2, and YY1 was a significant hit because of its high binding affinity.

YY1 is a common transcription factor and is overexpressed in a variety of cancers. YY1 is involved in the transcription of a variety of oncogenes and it, thus, participates in the regulation of cell cycle, apoptosis, and metastasis of cancer cells [30]. Through database screening, we found that YY1 and EMI2 may have a regulatory relationship. We, therefore, used a dual-luciferase reporter assay to predict the binding of YY1 and EMI2. We found that YY1 promoted the transcription of EMI2. YY1 competitively bound to *Rb* gene to cause the cell cycle to enter the S phase and promote the proliferation of tumor cells [31]. However, when YY1 was silenced, the cell cycle was arrested in the G1 phase, which was consistent with previous reports. However, after silencing YY1 and EMI2, the cell cycle was arrested in the G1 phase and the number of early apoptotic cells increased. This suggested that YY1 may activate EMI2 to promote cell cycle progression and inhibit apoptosis in bile duct cancer cells.

EMI2 is an inhibitor of APC/C, which is involved in regulating the progression of cell cycle-related proteins [32]. To explore the influence of EMI2, we detected the changes in certain molecules that are involved in 19 different signaling pathways in stable transgenic RBE cells after silencing EMI2, and the expression of CDKN1B and P53 was found to be increased. CDKN1B is downstream of the PI3K/Akt signaling pathway [33], and we speculated that EMI2 may play a role in the regulation of the PI3K/Akt signaling pathway. Western blotting results showed that the expression levels of PI3K, Akt, and MDM2 decreased after EMI2 was silenced, and MDM2 inhibited the activation of P53 [34]. Therefore, we speculated that EMI2 might affect PI3K/Akt signaling to regulate the cell cycle progression of bile duct cancer cells. However, YY1 directly activated the phosphorylation of Akt [35], thus, activating the PI3K/Akt signaling pathway. Therefore, we speculated that YY1 transcriptionally activated the expression of EMI2 and promoted the progression of bile duct carcinoma through the PI3K/Akt signaling pathway.

Our study had certain limitations. First, we did not verify whether EMI2 bound to CDKN1B. Secondly, we did not verify the effect of YY1 on the proliferation and metastasis of bile duct carcinoma in vivo. We will address these issues in future studies.

In conclusion, we found that EMI2, as an oncopromoter, promotes the progression of CCA. YY1 activates EMI2 and promotes PI3K/AKT/P27 signaling, thus, increasing the proliferation and metastasis and inhibiting the apoptosis in bile duct carcinoma. These results provided a novel target to study the molecular mechanism underlying CCA.

## Supplementary Information

**Additional file 1: Table S1.** Supplementary form text description. **Table S2.** qPCR Primers used for detecting miRNAs expression. **Table S3.** Primary antibodies used in this study. **Table S4.** Clinical information in the TME chip. **Table S5.** Sequencing of Wild type and mutant FBXO43.

**Additional file 2: Figure S1.** Bioinformatics analysis and TME microarray expression of EMI2. **Figure S2.** Fluorescence detection of the transfection efficiency of EMI2 silencing. **Figure S3.** Downstream pathway screening after EMI2 silencing. **Figure S4.** The changes of EMI2 and YY1 in HIBEpiC and HUCCT1 cells after oeEMI2.

## Data Availability

The raw datasets generated during the current study will be made available by the corresponding author upon reasonable request.
