# Supplementary material for: YY1 activates EMI2 and promotes the progression of cholangiocarcinoma through the PI3K/Akt signaling axis
Source: Cancer Cell Int. 2021 Dec 21;21:699. doi: 10.1186/s12935-021-02328-6 (PMC8693494; doi:10.1186/s12935-021-02328-6)
Supplement: Supplementary file 2 — Additional file 2: Figure S1. Bioinformatics analysis and TME microarray expression of EMI2. Figure S2. Fluorescence detection of the transfection efficiency of EMI2 silencing. Figure S3. Downstream pathway screening after EMI2 silencing. Figure S4. The changes of EMI2 and YY1 in HIBEpiC and HUCCT1 cells after oeEMI2. [file 12935_2021_2328_MOESM2_ESM.doc]

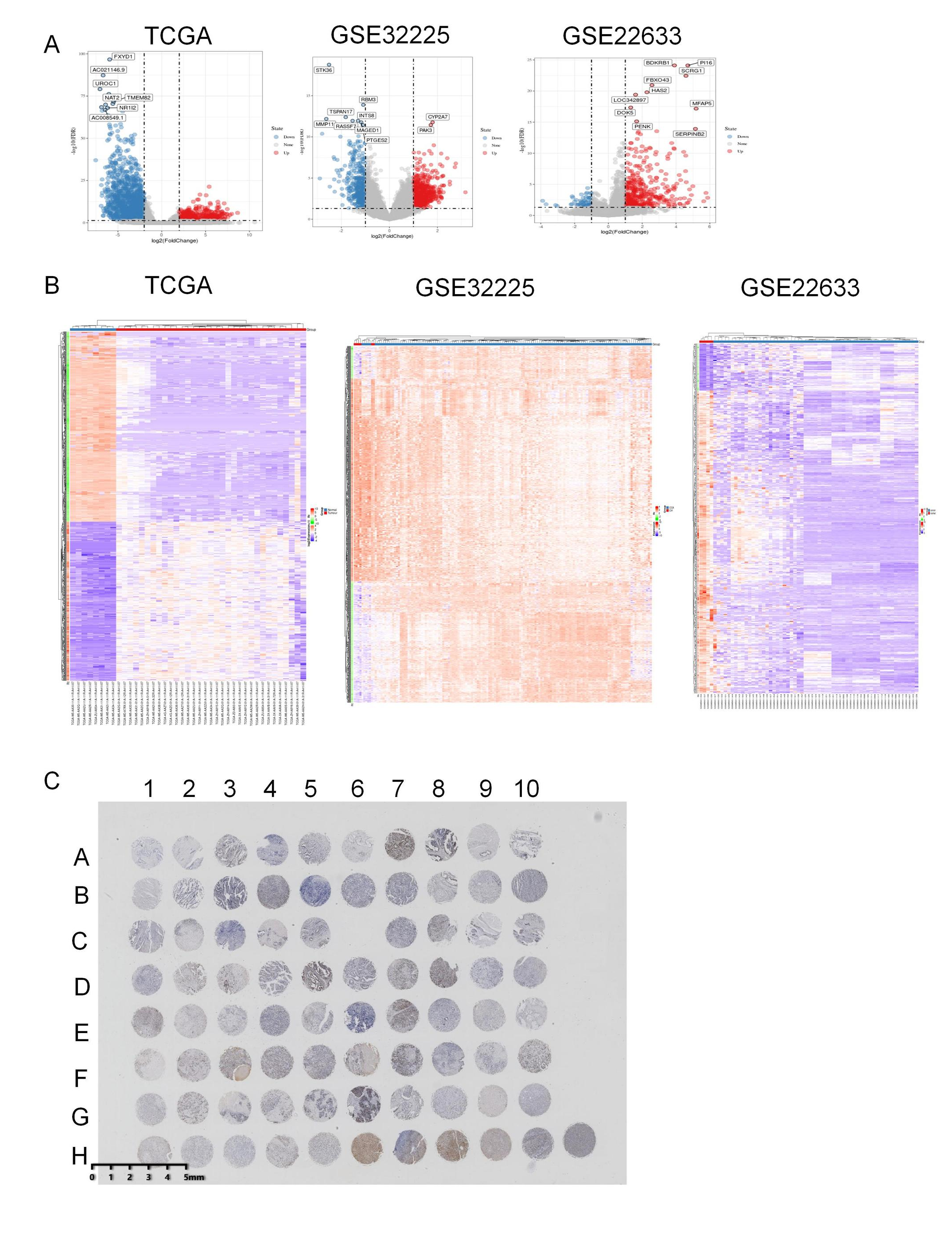


Figure S1 Bioinformatics analysis and TME microarray expression of EMI2. A. TCGA, GSE3225 and GSE22633, and the top 10 genes were selected as markers. B. TCGA, GSE3225 and GSE22633 differential gene volcano map; C. The expression of EMI2 in TME chip was detected by immunohistochemistry.


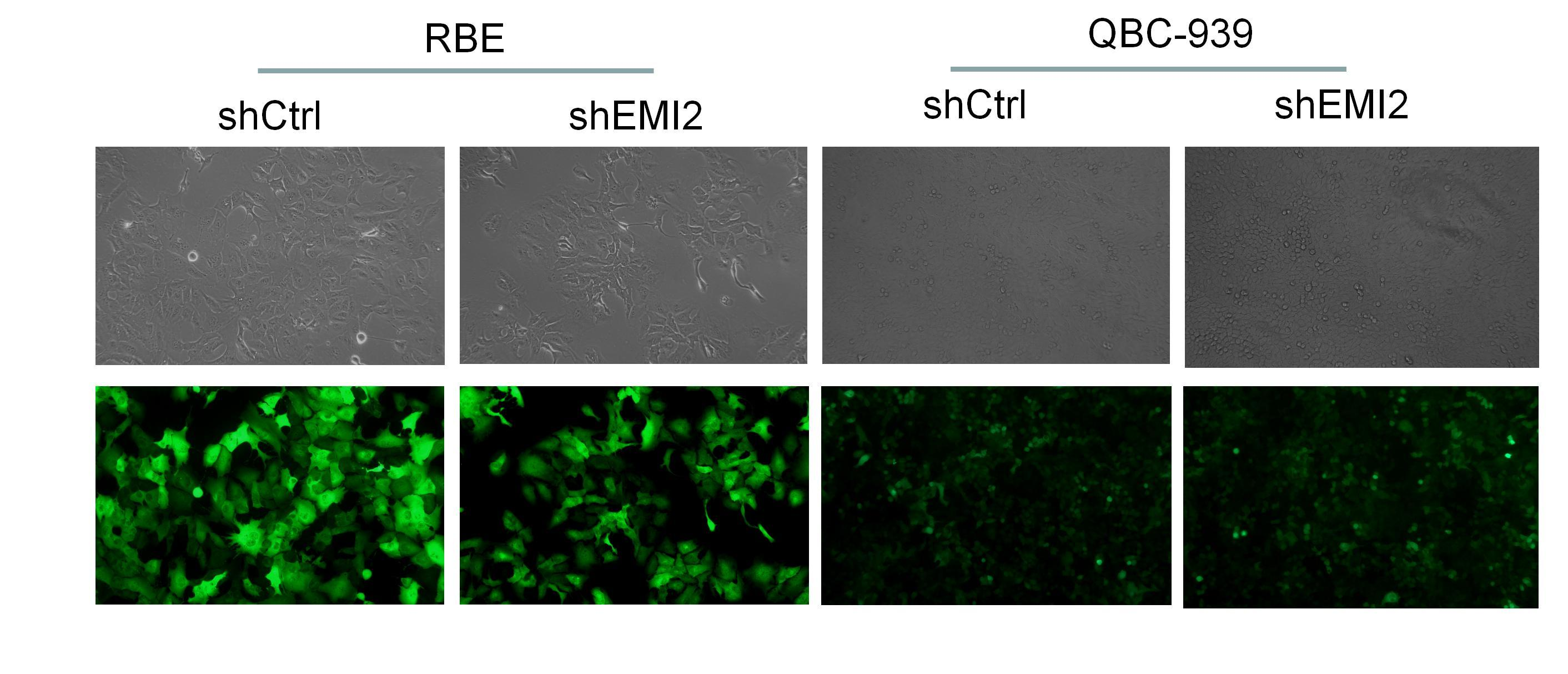


Figure S2 Fluorescence detection of the transfection efficiency of EMI2 silencing.


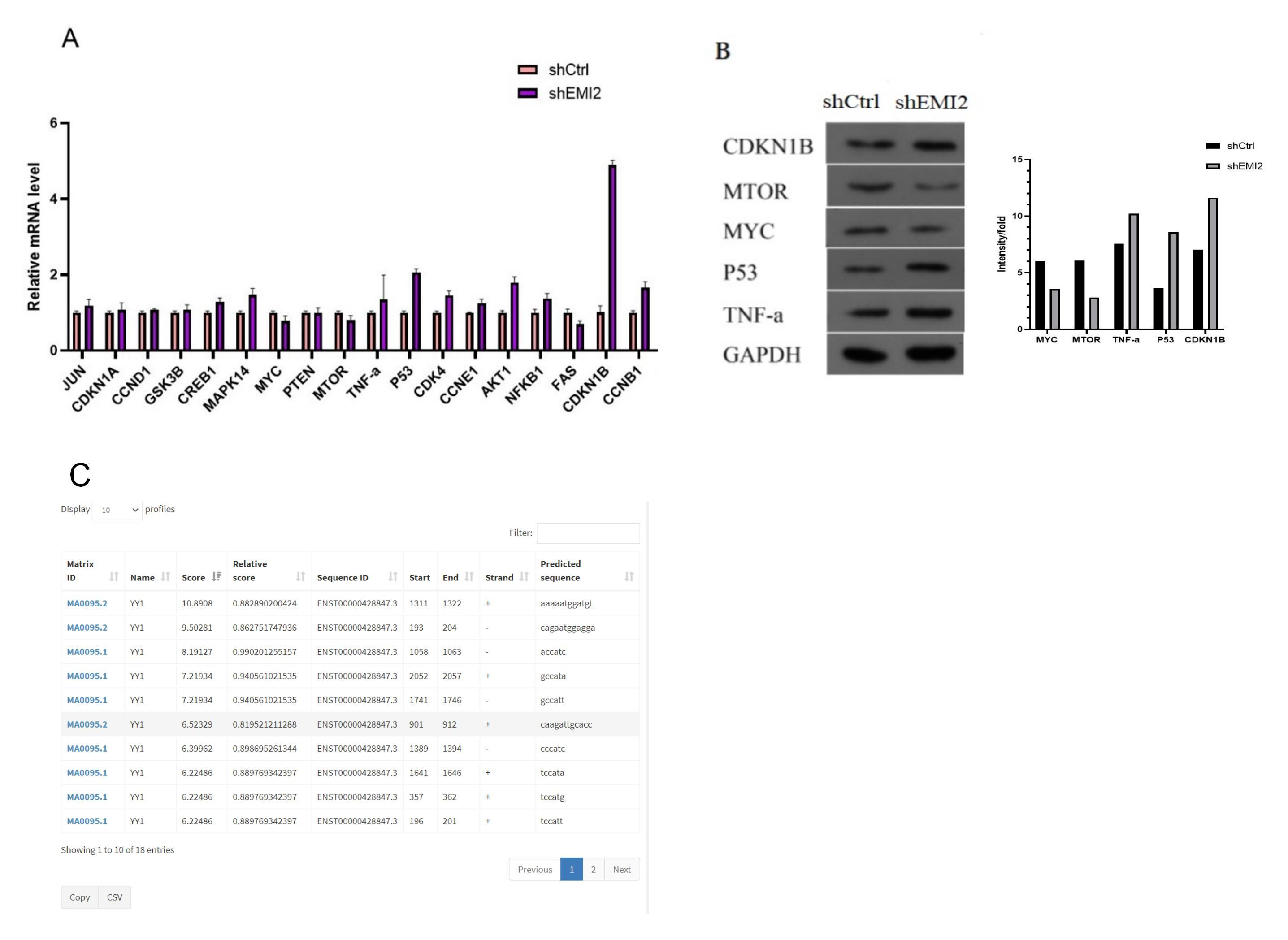


Figure S3 A. The mRNA levels of 19 star molecules in RBE cells silenced by EMI2 were detected by RT-PCR. B.Western blot assay verified the protein level changes of the 5 genes with the biggest differences; C. Prediction of binding sites of YY1 and EMI2 in ASPAR database(*, *p*＜0.05）.


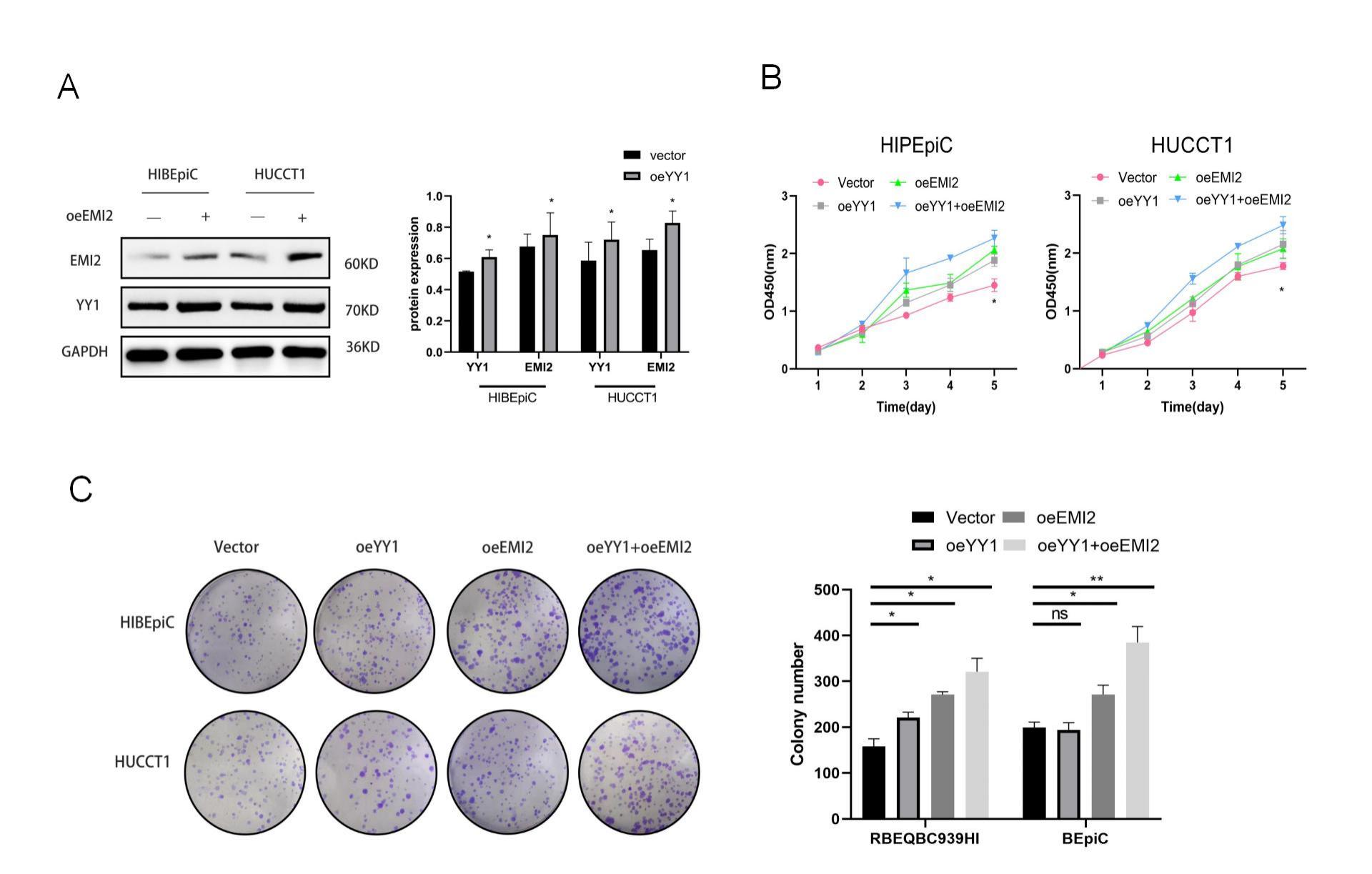


Figure S4 A. The changes of EMI2 and YY1 in HIBEpiC and HUCCT1 cells after oeEMI2 were detected by Western blot.; B.The change of OD450 in HUCCT1 and HIBEpiC cells were detected by CCK-8 assay; C. Clone experiment to detect value-added (*, *p*＜0.05）.
